# Screening for anti-influenza virus compounds from traditional Mongolian medicine by GFP-based reporter virus

**DOI:** 10.3389/fcimb.2024.1431979

**Published:** 2024-07-12

**Authors:** Mao-Shun Nie, Xiao-He Li, Sen Zhang, Dan-Dan Zeng, Yu-Rong Cai, Da-Xin Peng, Tao Jiang, Jian-Ping Shi, Jing Li

**Affiliations:** ^1^ College of Veterinary Medicine, Yangzhou University, Yangzhou, China; ^2^ College of Basic Medical Sciences, Inner Mongolia Medical University, Hohhot, China; ^3^ State Key Laboratory of Pathogen and Biosecurity, Academy of Military Medical Sciences, Beijing, China; ^4^ College of Traditional Chinese Medicine, Inner Mongolia Medical University, Hohhot, China

**Keywords:** GFP-based reporter virus, influenza A virus, traditional mongolian medicine, kaempferide, Curcumin, Cardamonin

## Abstract

**Introduction:**

Screening for effective antiviral compounds from traditional Mongolian medicine not only aids in the research of antiviral mechanisms of traditional medicines, but is also of significant importance for the development of new antiviral drugs targeting influenza A virus. Our study aimed to establish high-throughput, rapid screening methods for antiviral compounds against influenza A virus from abundant resources of Mongolian medicine.

**Methods:**

The use of GFP-based reporter viruses plays a pivotal role in antiviral drugs screening by enabling rapid and precise identification of compounds that inhibit viral replication. Herein, a GFP-based reporter influenza A virus was used to identify potent anti-influenza compounds within traditional Mongolian medicine.

**Results:**

Our study led to the discovery of three active compounds: Cardamonin, Curcumin, and Kaempferide, all of which exhibited significant antiviral properties *in vitro*. Subsequent analysis confirmed that their effectiveness was largely due to the stimulation of the antiviral signaling pathways of host cells, rather than direct interference with the viral components, such as the viral polymerase.

**Discussion:**

This study showcased the use of GFP-based reporter viruses in high-throughput screening to unearth antiviral agents from traditional Mongolian medicine, which contains rich antiviral compounds and deserves further exploration. Despite certain limitations, fluorescent reporter viruses present substantial potential for antiviral drug screening research due to their high throughput and efficiency.

## 1 Introduction

The relentless pursuit of targeted therapies is crucial in the global health landscape, where respiratory pathogens still poses a persistent threat (Hedberg et al., 2023; Song et al., 2023). Avian influenza A viruses (IAV) such as H7N9 (Deng et al., 2017) and H5N1 (He et al., 2010; Sun H. et al., 2014), as well as adenoviruses (Liu et al., 2014), continue to pose sporadic outbreaks, posing an ongoing threat to human health. Additionally, artificial intelligence (AI) models predict a high risk of IAV and coronaviruses infecting humans after cross-species transmission (Li et al., 2020; Li J. et al., 2022). The identification and evaluation of antiviral drugs are fundamental to the treatment of viral infections and the management and prevention of epidemic. Given this, research into the development of new antiviral drugs for IAV remains of significant practical importance. Initially, antiviral strategies targeted the IAV’s M2 ion channel protein using drugs like amantadine (Calderon. et al., 2019) and rimantadine (Scott. et al., 2020). These early interventions aimed to impede the virus’s ability to infect host cells, but the rise of resistant viral strains rendered them less effective. This challenge led to the development and widespread adoption of neuraminidase inhibitors, including Oseltamivir (Tamiflu) (M, 2004)and Zanamivir (Relenza) (Hayden. et al., 2000). These drugs function by blocking a critical enzyme, neuraminidase, which is necessary for the virus to exit infected cells and spread the infection further. However, the influenza virus’s capacity for mutation demands continual innovation in antiviral drug development. Baloxavir marboxil represents the latest advancement in this endeavor, targeting the virus’s polymerase complex to thwart its replication process (Hayden et al., 2018). By inhibiting the cap-dependent endonuclease function, Baloxavir impairs the synthesis of viral mRNA, a key step in the production of viral proteins and the assembly of new virus particles. Ongoing research is focused on discovering antivirals with broad-spectrum efficacy, capable of targeting multiple IAV strains and reducing the likelihood of resistance development. These efforts are vital in establishing a more robust defense against the ever-evolving IAV and ensuring preparedness for future flu seasons and potential pandemics. While the development of emerging AI technology enables rapid phenotypic predictions such as viral adaptability and the swift virtual screening of antiviral drugs (Popov et al., 2023), the key to antiviral drug research still lies in the high-throughput and rapid experimental assessment of antiviral activity of target drugs. Traditional virological experimental screenings for antivirals are often time-consuming. What’s more, drugs screened based on key enzymatic experiments related to viral proliferation, such as RNP polymerase, often lack reliability. The main reasons include that targeting specific enzymes does not necessarily equate to antiviral activity, or drugs targeting non-enzymatic sites are prone to be overlooked. Considering these, the development of high-throughput screening methods for antiviral drugs is not only a scientific challenge but also a necessity for maintaining global health security.

With the development of globalization and modern technology, many traditional medical systems are continuously integrating with modern medicine, contributing to the advancement of human health and medicine. Traditional medical systems from various ethnic groups around the world are also gradually becoming a treasure trove for screening anti-infection and antiviral drugs (Ye. et al., 2023). Steeped in the rich cultural heritage of Mongol nationality, traditional Mongolian medicine is an important part of Chinese traditional culture, which offers a holistic approach to healthcare, integrating herbal remedies, acupuncture, and moxibustion, and has been a cornerstone of healing practices for centuries (Meng et al., 2009; Song et al., 2022; Li. et al., 2023). This ancient system has continuously evolved, incorporating the indigenous healing wisdom of the Mongolian people while also absorbing therapeutic concepts from Tibetan, Chinese, and Indian medicinal traditions (Li X. et al., 2022; Long et al., 2022). A significant aspect of this traditional medicine is the use of various herbs that have shown promising antiviral properties in preliminary research. Notably, Rhodiola rosea, commonly referred to as “Golden Root”, is recognized for its antiviral effects, particularly against influenza A virus (IAV) (Jeong et al., 2009; Döring et al., 2022) and herpes viruses (Tettevi et al., 2022), suggesting its potential in combating viral infections. Likewise, extracts from other medicinal plants, such as Schizandra chinensis (Qi et al., 2023) and Glycyrrhiza uralensis (Adianti et al., 2014), have been identified to possess antiviral capabilities. These botanical compounds are key to traditional Mongolian medicine and are now being explored as potential foundations for new antiviral therapies. Current scientific investigations are delving into the molecular structures and actions of these herbal extracts, aiming to unveil their potential as innovative sources for antiviral drug development, thereby bridging ancient practices with modern medical science. However, how to achieve high-throughput screening of antiviral compounds from traditional medicines is currently a pressing technological challenge.

Fluorescent reporter viruses have revolutionized the field of antiviral drug screening by providing a powerful tool for rapid and efficient evaluation of therapeutic agents (Cherkashchenko et al., 2023; Zhang et al., 2024). These specially designed viruses incorporate genes for fluorescent proteins that are expressed during viral replication. Upon infection of host cells, these viruses produce a fluorescent signal that can be easily detected and measured. The brilliance of the emitted light serves as a direct indicator of the level of viral replication, allowing researchers to swiftly gauge the potency of antiviral drugs (Manicassamy et al., 2010; Zhang et al., 2020). The use of these reporter viruses significantly expedites the drug discovery process. By offering a high-throughput screening capability, they enable the simultaneous testing of numerous compounds, drastically reducing the time and resources required, compared to conventional methods such as plaque assays or cytopathic effect-based assays. Moreover, the real-time nature of the fluorescent readout allows for dynamic monitoring of viral growth and inhibition, providing insights into the kinetics of antiviral action (Pahmeier et al., 2021). This technology is highly adaptable, fitting seamlessly into automated platforms that can handle large-scale drug screenings with minimal human intervention, further enhancing the workflow efficiency. The precision and speed with which fluorescent reporter viruses can deliver results have made them an indispensable asset in the race to identify new antiviral drugs, particularly in the face of emerging viral threats where rapid response is crucial, such as SARS-CoV-2 (Chiem et al., 2022; Yang et al., 2023). Their application extends beyond initial screenings, contributing to subsequent optimization and characterization of promising antiviral candidates, ultimately accelerating the path from laboratory discovery to clinical application. The technology based on fluorescent reporter viruses will be an effective method for high-throughput screening of antiviral compounds in traditional medicine.

Here, we utilized the GFP-based reporter IAV (GFP-IAV) to conduct *in vitro* screening of active compounds in traditional anti-IAV Mongolian medicine, and successfully identified three compounds with anti-IAV activity. Subsequent validation of their anti-IAV effects was performed, and their potential antiviral mechanisms were explored. Our research represented an initial application of screening antiviral compounds using the GFP-IAV, laying the groundwork for subsequent high-throughput screening of antiviral components in traditional medicines.

## 2 Materials and methods

### 2.1 Cell and viruses

MDCK (Madin-Darby canine kidney) cells (ATCC, Manassas, VA, USA, CCL-34), and HEK293T (Human embryonic kidney) cells (ATCC, CRL-3216) were cultured at 37°C/5% CO_2_ in Dulbecco’s modified Eagle’s medium (DMEM, Thermo Fisher Scientific, Waltham, MA, USA) supplemented with 10% heat-inactivated fetal bovine serum (Gibco) and 1% PSG (100 U/mL penicillin, 100 μg/mL streptomycin). A549 (Human lung epithelial carcinoma) cells (ATCC, CCL-185) were cultured at 37°C/5% CO_2_ in F-12K Nutrient Mixture (F-12K, Thermo Fisher Scientific) supplemented with 10% heat-inactivated fetal bovine serum (Gibco) and 1% PSG (100 U/mL penicillin, 100 μg/mL streptomycin). H1N1 subtype IAV A/California/California/07/2009 (CA07) were propagated in MDCK cells.

### 2.2 Reverse genetics rescue of GFP-IAV

GFP-IAV was rescued by reverse genetics techniques as described in our previous study (Sun W. et al., 2014). Briefly, the GFP coding sequence (with stop codon) was linked to the NA segment (without stop codon) of CA07 via a 2A peptide, then incorporating a fragment of the NA segment 3’ end sequence post-GFP as the IAV polymerase recognition signal. The linked fragments were cloned into pHW2000 plasmids, and the constructs were confirmed by complete sequencing to ensure the absence of unwanted mutations. 293T cells were transfected with the constructed NA-GFP and the other 7 wild-type fragments using Lipofectamine 3000 transfection reagent (Thermo Fisher Scientific) in 24-well plates. The supernatant of the transfected cells was collected 24 hour (h) post-transfection and transferred to MDCK cells with infection medium (6-well plates). The supernatant with rescued viruses was collected at 72 h post-infection, and then the virus was propagated again in MDCK cells. Recombinant viruses (GFP-IAV) were titered by plaque assay.

### 2.3 Screening for anti-IAV compounds by GFP-IAV

MDCK cells were cultured in 24-well plates. After the cell density reached 100% confluence, the cells were washed three times with PBS and incubated with GFP-IAV at the multiplicity of infection (MOI) of 0.001. Next, cells were washed once with PBS and cultured at 37°C/5% CO_2_ in DMEM medium supplemented with 2 μg/mL TPCK-treated trypsin and without FBS. Meanwhile, the medium was added with the compounds that awaiting screening (final concentration of 50 μM and 25 μM). The cell status and GFP fluorescence intensity was observed at 12, 18, 24 h post-infection, and the images were captured using an Olympus Fluorescence IX73 Microscope.

### 2.4 Quantitative real-time PCR

Total RNA was extracted from virus culture supernatant using a PureLink RNA Mini Kit (Thermo Fisher Scientific). QRT-PCR was performed using an One Step TB Green PrimeScript PLUS RT-PCR Kit (TaKaRa, Shiga Prefecture, Kusatsu City, Japan) with a Light Cycler 480 II (Roche, Basel, Switzerland). Virus titer was determined by qRT-PCR and converted to pfu numbers by standard curve.

### 2.5 Plaque assay

MDCK cells were cultured in 12-well plates. After the cell density reached 100% confluence, cells were infected with IAV at 37°C. After 1 h of virus adsorption, cells were overlaid with DMEM containing 0.8% Low Melting Point agarose and TPCK-treated trypsin (2 μg/mL) and then incubated at 37 °C for 72 h. At 72 h post-infection, cells were fixed for 30 min at room temperature with 4% paraformaldehyde. The agarose overlay was removed and stained for 15 min with 1% crystal violet in methanol. The number of plaques in each well was counted.

### 2.6 Transcriptomic sequencing analysis

The A549 cells were cultured in 6-well plates. After the cell density reached about 80–90%, cells were washed three times with PBS and the cell culture medium was replaced with serum-free fresh medium containing different compounds (Kaempferide, Curcumin and Cardamonin) at concentration of 50 μM, with DMSO as negative control. Total RNA was extracted with TRIzol^®^ Reagent (Thermo Fisher Scientific) and transcriptomic sequencing analysis was conducted by Novogene (Beijing, China P.R.). The Kyoto Encyclopedia of Genes and Genomes (KEGG) pathway database under biological process (BP), cellular component (CC) and molecular function (MF) categories were used for functional annotation and enrichment analyses. The bubble chart for bioinformatics-related data analysis was plotted by http://www.bioinformatics.com.cn (accessed on 23 December 2023), an online platform for data analysis and visualization (Luo and Brouwer, 2013).

### 2.7 Detection of polymerase activity

293T cells were cultured in 24-well plates. After the cell density reached about 80% confluence, cells were co-transfected with pHW2000 plasmids encoding PB2, PB1, PA, and NP proteins, pHH21 plasmid expressing negative vRNA-like firefly luciferase (Fluc) RNA and plasmid encoding renilla luciferase (Rluc) as internal reference using Lipofectamine 3000 reagent (Thermo Fisher Scientific). At 6 h post-transfection, cells were washed three times with PBS and the cell culture medium was replaced with fresh medium containing different compounds (Kaempferide, Curcumin and Cardamonin) at different concentrations (25 μM, 12.5 μM, 6.25 μM and 3.125 μM) with DMSO as negative control. After 24 h of incubation with the compounds, cells were harvested and lysed. The polymerase activity was analyzed using a Dual-Luciferase Reporter Assay System kit (Promega, Madison, WI, USA). All assays were performed independently and in triplicate.

### 2.8 Western blot analysis

Cells were lysed with RIPA lysate containing protease inhibitors to extract proteins. Protein concentration was quantified using the BCA assay. The protein was diluted to a final concentration of 0.2 μg/μL, DTT, 5×MAster Mix, and Ladder were prepared according to the manufacturer’s instructions, and NP primary Antibody was diluted 1:250 with Antibody Diluent II. Luminescent solution was prepared by mixing equal volumes of Lumino-S and Peroxide, Anti-Rabbit Secondary Antibody was used for Secondary Antibody, and samples were loaded in sequence. The JessTM automatic protein expression analysis system was used to detect protein bands.

### 2.9 Statistical analysis

The GraphPad Prism 9.0 software was used to conduct all statistical analysis. Data were presented as means ± SD of at least three independent experiments. Differences between the two groups were evaluated using Student’s *t*-test, and differences between three or more groups were evaluated using analysis of variance (ANOVA). statistical significance was set at *p* < 0.05.

## 3 Results

### 3.1 Workflow for screening anti-IAV compounds using GFP-based reporter virus

**Figure 1:**
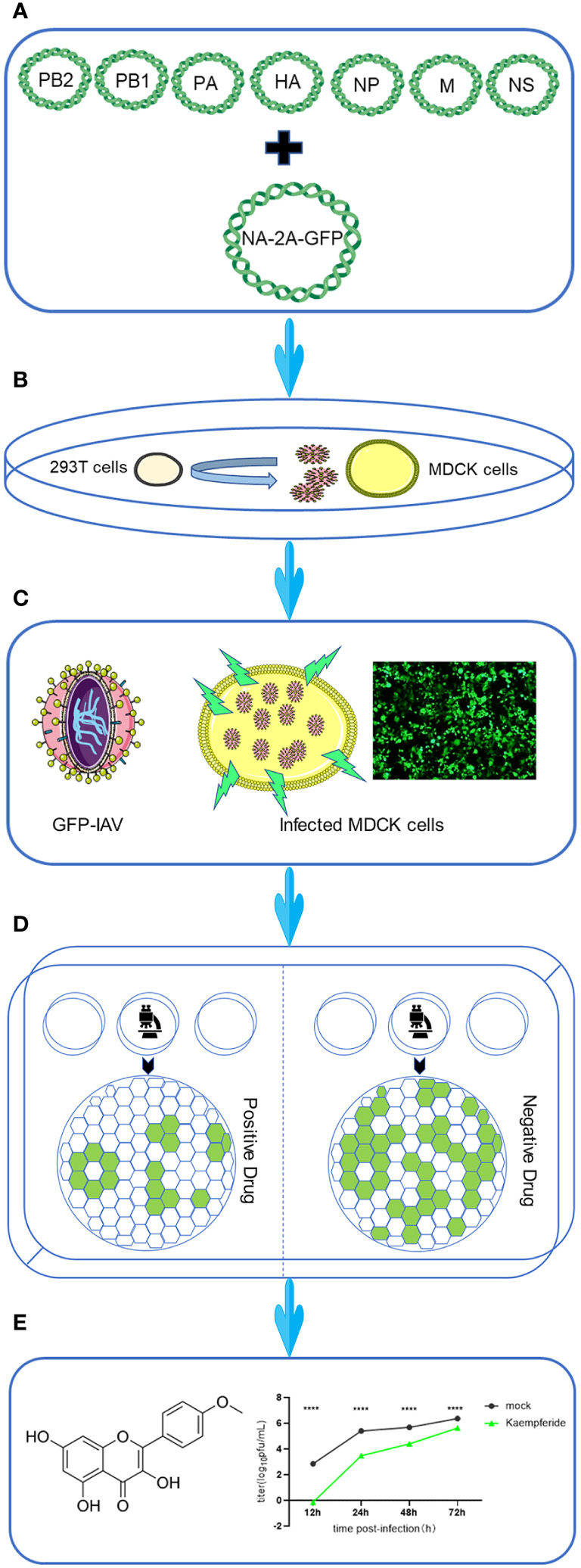
Workflow for screening anti-IAV compounds using GFP-based reporter virus. The workflow was designed as followed. **(A)** Eight-Plasmid Schematic of GFP-IAV reverse genetics. **(B)** 293T cells were transfected with eight engineered plasmids, and the viruses harvested at 48 h post-transfection were subsequently inoculated into MDCK cells to be further amplified for a 72-hour duration. **(C)** The schematic diagram of GFP-IAV. GFP fluorescence could be detected when MDCK cells were infected with GFP-IAV. **(D)** Cells infected with GFP-IAV were treated with candidate anti-viral drugs. The expression of GFP would be reduced if the drug exhibited antiviral activity against IAV; conversely, higher expression GFP levels indicated a lack of antiviral efficacy. **(E)** Following the screening process, drugs with antiviral activity were subjected to further experimental validation. Statistical significance was calculated using two-way ANOVA (****p < 0.0001).

As shown in the workflow, the coding sequence of enhanced Green Fluorescent Protein (eGFP) was linked to the NA sequence of IAV via the 2A peptide (**Figure 1A**). Then, the full length of all eight segments of IAV, including NA-GFP, were amplified from viral RNA segment by RT-PCR and constructed into the pHW2000 vectors (**Figure 1A**). Recombinant viruses were produced by transfecting 293T cells with eight engineered plasmids, and the viruses harvested after 48 h were subsequently inoculated into MDCK cells to be further amplified for a 72-h duration (**Figure 1B**). A substantial quantity of IAV carrying the GFP reporter gene (GFP-IAV) could be obtained from the supernatant of MDCK cells (**Figure 1C**). Upon infecting MDCK cells with the GFP-IAV, GFP could be observed under a fluorescence microscope as early as 12 h post-infection, and prominent fluorescence would be noticeable at approximately 18 h post-infection (**Figure 1C**). Thus, the GFP-IAV coul be utilized for the rapid screening of antiviral drugs against IAV. The candidate drugs were incubated with MDCK cells infected by the GFP-IAV, and after approximately 12–18 hs, the expression levels of GFP could be observed. Compare the GFP levels with a negative control, the expression of GFP would be reduced if the drug exhibited antiviral activity against IAV; conversely, higher expression GFP levels indicated a lack of antiviral efficacy (**Figure 1D**). By GFP-IAV, high-throughput screening of antiviral drugs could be rapidly achieved. The drugs obtained after screening were further identified by measuring the influenza virus proliferation curve, resulting in a high positivity rate (**Figure 1E**).

### 3.2 The construction and biological identification of the GFP-IAV

**Figure 2:**
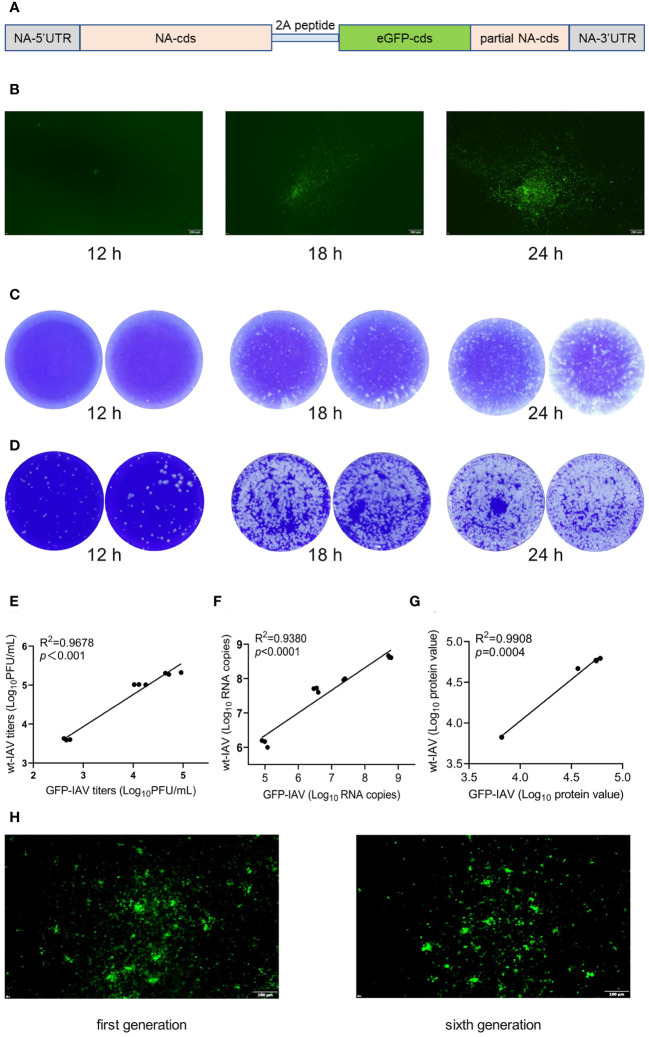
The construction and biological identification of the GFP-IAV. **(A)** The plasmid construction schematic of NA-2A-GFP. **(B)** MDCK cells were infected with the GFP-IAV and the expression of GFP was observed at 12, 18, and 24 h post-infection using a fluorescence microscope. **(C, D)** MDCK cells were infected with GFP-IAV **(C)** and wild-type IAV **(D)** (moi=0.001) and the supernatant was collected at 12, 18, and 24 h post-infection. The viral titers were then determined by plaque assay. **(E–G)** Correlation analysis of the titers **(C)**, RNA copy numbers **(D)** and protein expression **(E)** between wild-type IAV and GFP-IAV at different time points during their proliferation process. **(H)** GFP expression of GFP-IAV after the first generation and six consecutive passages.

2A peptides are short sequences of amino acids used in molecular biology to mediate the co-expression of multiple proteins from a single mRNA. This “ribosome skipping” technology allows for the production of separate proteins from the same transcript without the need for additional promoters or multiple cloning sites. The influenza A virus strain A/California/07/2009 (CA07) served as the scaffold, linking the GFP coding sequence (with stop codon) to the NA segment (without stop codon) via a 2A peptide, and incorporating a fragment of the NA segment 3’ end sequence post-GFP as the IAV polymerase recognition signal (**Figure 2A**). Subsequently, a recombinant influenza virus encapsulating the GFP encoding gene, GFP-IAV, was successfully obtained through eight-plasmid reverse genetics operations (**Figure 1B**). To verify the expression efficiency of GFP during GFP-IAV infection, MDCK cells were infected with the virus and the expression of GFP was observed at 12, 18, and 24 h post-infection using a fluorescence microscope. The results indicated that faint fluorescence could be observed at 12 h post-infection, with more pronounced fluorescence visible after 18 h, often before any significant cytopathic effects (CPE) are apparent (**Figure 2B**). By 24 h post-infection, the fluorescence become even more distinct (**Figure 2B**). It demonstrated that the recombinant GFP-IAV could effectively infect MDCK cells and express GFP, allowing for the assessment of viral infection prior to the appearance of CPE. We then compared the replication capabilities of the recombinant GFP-IAV with that of the wild-type IAV virus. MDCK cells were infected with two different viruses (moi=0.001) and the supernatant was collected at 12, 18, and 24 h post-infection. The viral titers were then determined by plaque assay. The plaque assay results indicated that under the same infection conditions, the progeny virus particles produced by the recombinant GFP-IAV (**Figure 2C**) were fewer than those produced by the wild-type virus (**Figure 2D**). This suggested that the replication efficacy of the recombinant GFP-IAV was diminished in comparison to the wild-type counterpart, which could be due to the additional burden of carrying the GFP gene, resulting in a reduced capacity for propagation. To further evaluate whether GFP-IAV accurately mirrors wild-type IAV at the RNA, protein, and viral levels, correlation analyses between wild-type IAV and GFP-IAV were conducted at various infection time points, including viral titers (**Figure 2E**; **Supplementary Figure 1A**), RNA copy number (**Figure 2F**; **Supplementary Figure 1B**), and protein expression (**Figure 2G**; **Supplementary Figure 1C**). The correlation coefficient between wild-type IAV and GFP-IAV were 0.96 for viral titers, 0.93 for RNA copy numbers, and 0.99 for protein level. The results showed that GFP-IAV could reflect the biological characteristics of wild-type IAV to a certain extent. Then, to further confirm the genetic stability of the GFP-IAV, it was passaged continuously for 6 generations on MDCK cells. The results showed that the expression of GFP during infection after sixth passages was essentially consistent with the initial passage (**Figure 2H**), indicating that the GFP gene has not been lost. In conclusion, recombinant GFP-IAV could infect MDCK cells and proliferate effectively, with the amount of GFP expression increasing over time post-infection. And the GFP gene was not easily lost during the process of viral passage. Observable fluorescence preceded significant CPE, indicating that GFP-IAV could be used for the rapid screening of antiviral drugs against IAV.

### 3.3 Three Mongolian medicine compounds with antiviral activity identified by GFP-IAV

**Figure 3:**
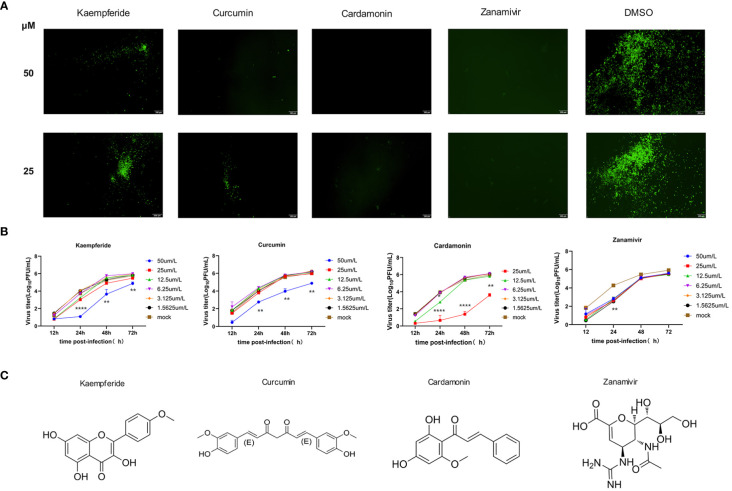
Three Mongolian medicine compounds with antiviral activity identified by GFP-IAV. **(A)** MDCK cells were infected with GFP-IAV and added natural compounds (Kaempferide, Curcumin and Cardamonin) to the supernatant at concentration of 50 μM and 25 μM, with Zanamivir as positive control and DMSO as negative control. GFP fluorescence was observed at 24 h post-infection. **(B)** MDCK cells were infected with GFP-IAV and added natural compounds (Kaempferide, Curcumin and Cardamonin) to the supernatant at different concentrations of 50 μM, 25 μM, 12.5 μM, 6.25 μM, 3.125μM and 1.5625 μM, with Zanamivir as positive control and DMSO as negative control. Virus titer was determined by qRT-PCR at different time points (12 h, 24 h, 48 h and 72 h post-infection) and converted to pfu numbers by standard curve. Virus growth curves were examined. **(C)** Chemical structures of Kaempferide, Curcumin, Cardamonin and Zanamivir. Statistical significance was calculated using two-way ANOVA (*p < 0.05; **p < 0.01; ****p < 0.0001).

To select effective anti-IAV compounds in traditional Mongolian medicine, traditional Mongolian formulations known for their antiviral activity were reviewed. The natural compounds within these formulations were organized and 18 natural compounds were selected as potential candidates (**Supplementary Table 1**). Since most compounds exhibited high cytotoxicity at 200 uM and 100 uM, the maximum compound concentration was set at 50 uM for the subsequent screenings. MDCK cells were infected with GFP-IAV and added natural compounds to the supernatant at concentration of 50 μM and 25 μM, with Zanamivir as positive control and DMSO as negative control. GFP fluorescence was continuously observed at 12 h post-infection. The results indicated that at 24 h post-infection, cells treated with Kaempferide, Curcumin and Cardamonin demonstrated significantly lower GFP fluorescence intensity compared to the DMSO group at both concentration of 50 μM and 25 μM, additionally, the positive control group displayed a substantially diminished GFP fluorescence intensity as well (**Figure 3A**). It suggested that these three compounds might possess antiviral activity against IAV. To further verify their antiviral effects, proliferation curves of the GFP-IAV were conducted in MDCK cells treated with three different compounds and positive control compound at varying concentration gradients. The findings suggested that when exposed to elevated levels of the compounds (50 μM), the virus’s replicative efficacy was markedly decreased relative to the mock-treated group. Furthermore, as the compound’s concentration was lowered, the suppressive impact on viral replication correspondingly lessens (**Figure 3B**). Considering these, we believed that Kaempferide, Curcumin and Cardamonin identified through GFP-IAV screening were worth further investigation due to their potential anti-influenza virus activity. The chemical structures of these three compounds and the positive control were shown in **Figure 3C**.

### 3.4 Three compounds have significant inhibitory effects on the wild-type IAV

**Figure 4:**
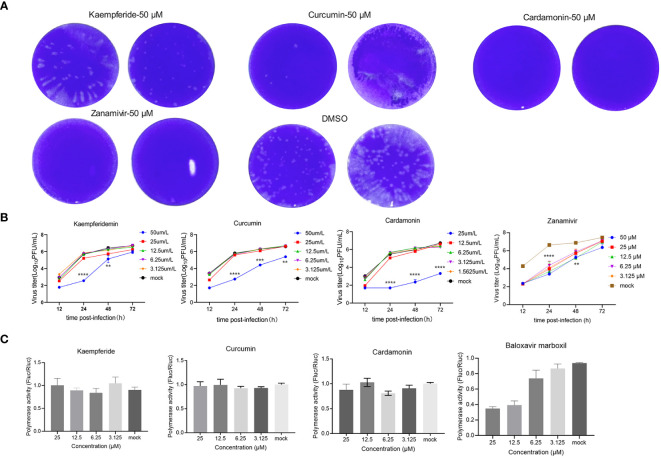
Three compounds have significant inhibitory effects on the wild-type IAV. **(A)** MDCK cells were infected with the wild-type IAV (moi=0.001) and added natural compounds (Kaempferide, Curcumin and Cardamonin) to the supernatant at concentration of 50 μM, with Zanamivir as positive control and DMSO as negative control. The IAV titers were determined through plaque assays at 48 h post-infection. **(B)** MDCK cells were infected with wild-type IAV and added natural compounds (Kaempferide, Curcumin and Cardamonin) to the supernatant at different concentrations of 50 μM, 25 μM, 12.5 μM, 6.25 μM and 3.125 μM, with Zanamivir as positive control and DMSO as negative control. Virus titer was determined by qRT-PCR at different time points (12 h, 24 h, 48 h and 72 h post-infection) and converted to pfu numbers by standard curve. Virus growth curves were examined. **(C)** 293T cells were co-transfected with pHW2000 plasmids encoding PB2, PB1, PA, and NP proteins, pHH21 plasmid expressing negative vRNA-like firefly luciferase (Fluc) RNA and plasmid encoding renilla luciferase (Rluc) as internal reference. The cells were then incubated with various concentrations (25 μM, 12.5 μM, 6.25 μM and 3.125 μM) of natural compounds (Kaempferide, Curcumin and Cardamonin), with Baloxavir marboxil as positive control and DMSO as negative control. Luciferase activity was measured at 24 h after transfection. Statistical significance was calculated using two-way ANOVA (**p < 0.01; ***p < 0.001; ****p < 0.0001).

To further verify the antiviral effects of these three compounds screened out by the GFP-IAV, MDCK cells were infected with the wild-type IAV (moi=0.001). After one-hour adsorption, the compounds were diluted in a two-fold concentration gradient and incubated with the infected cells. Supernatants were collected at 12, 24, 48 and 72 h post-infection, and the IAV titers were determined through plaque assays. The results of the plaque assay at 48 h post-infection of high-concentration compound treatment showed that the number of viral plaques for the three compounds and the positive control group were lower than that of the mock group (**Figure 4A**). The compounds’ effect on the proliferation efficiency of the wild-type IAV was essentially consistent with that of the GFP-IAV. It indicated that upon administration of the compounds at heightened concentrations (50 µM), there was a significant attenuation in IAV’s ability to replicate when compared to the mock-treated assemblage. Additionally, a reduction in the concentration of the compounds was associated with a proportional diminution in their inhibitory influence on viral propagation (**Figure 4B**). The IC_50_ of three compounds were calculated by the inhibition results on the IAV titers (**Supplementary Figure 2A**). To determine the cytotoxicity of these compounds, MDCK cells were treated with varying concentrations of each compound. After 24 hours, cell viability was measured and calculated the CC_50_ values for the three compounds (**Supplementary Figure 2B**). The SI values were showed in **Supplementary Table 2**. To further investigate whether the antiviral effects of the compounds were related to the inhibition of influenza virus polymerase activity, the influenza virus polymerase reporter system was used to measure the activity of the viral polymerase under compounds treatment. The Baloxavir marboxil was chosen as positive control due to its known inhibitory effects on the polymerase activity of influenza viruses. 293T cells were co-transfected with pHW2000 plasmids encoding PB2, PB1, PA, and NP proteins, pHH21 plasmid expressing negative vRNA-like firefly luciferase (Fluc) RNA and plasmid encoding renilla luciferase (Rluc) as internal reference. The cells were then incubated with various concentrations of compounds, and luciferase activity was measured at 24 h after transfection. Due to the poor adherence of 293T cells, detachment occurred at high concentration of 50 µM. Therefore, the maximum concentration was set at 25µM. The results indicated that at the highest concentration of 25 µM and under gradient dilution, the compounds did not exhibit significant inhibitory effects on the IAV polymerase, in contrast to the obvious inhibitory effects of the positive control group (**Figure 4C**). Taken together, these three compounds significantly inhibited the proliferation of the wild-type IAV, moreover, this inhibitory effect was independent of the IAV polymerase activity, suggesting that it might primarily affect the antiviral signaling pathways of the host cells.

### 3.5 Three compounds regulated the antiviral signaling pathways in host cells

**Figure 5:**
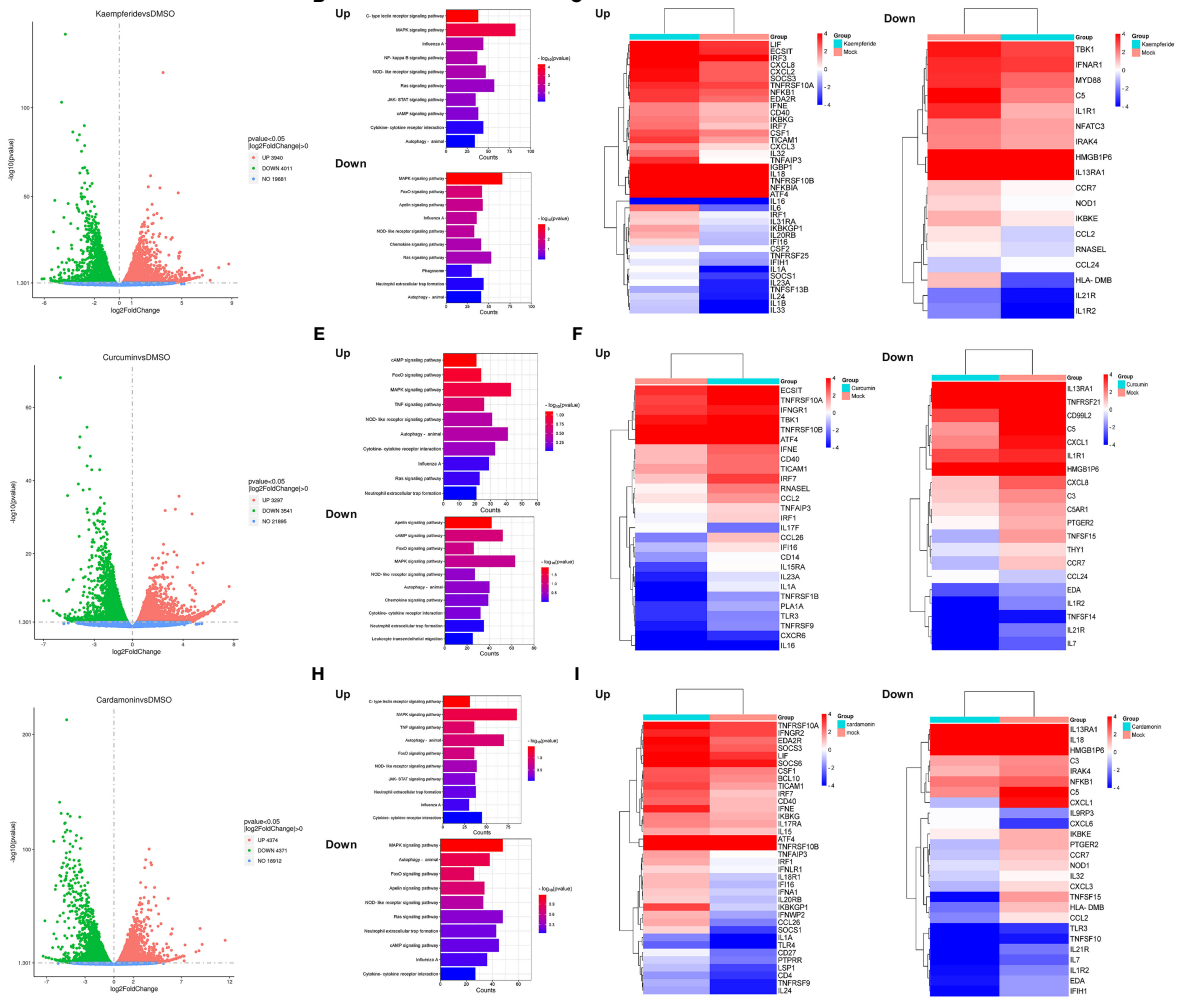
Three compounds regulated the antiviral signaling pathways in host cells. **(A, D, G)** Scatter plots showing the genes that were differentially expressed within the A549 cells after Kaempferide **(A)**, Curcumin **(D)** and Cardamonin **(G)** treatment, with DMSO as negative control. The red plots indicated the up-regulation genes, the green plots indicated the down-regulation genes and the grey plots indicated the un-changed genes. *P* value < 0.05 and |log2FoldChange| > 0. **(B, E, H)** KEGG pathways enrichment analysis of differential expressed genes associated with viruses after A549 cells treated with Kaempferide **(B)**, Curcumin **(C)** and Cardamonin **(D)**. **(C, F, I)** Heatmaps of genes enriched in the top ten up and down-regulated KEGG pathways enrichment analysis after A549 cells treated with Kaempferide **(C)**, Curcumin **(F)** and Cardamonin **(I)**. The colors depict the log2 fold change compared to the mock group. Each cell represents the average of the three samples.

To elucidate the antiviral mechanisms employed by these three compounds in combatting IAV, transcriptomic sequencing analysis was conducted to detect changes in cellular transcriptome after compounds treatment. A549 cells were either mock-treated with DMSO or treated with three compounds for 24 h, and the total RNA were purified for further transcriptomic sequencing. The results indicated that all three compounds have a significant impact on the expression of genes within A549 cells. After Kaempferide treatment, there were 3940 genes upregulated and 4011 genes downregulated in expression compared to the mock group (**Figure 5A**). Kyoto Encyclopedia of Genes and Genomes (KEGG) analysis further indicated that the major upregulated genes impacted by Kaempferide were associated with innate immunity-related pathways, including the NF-kappa B signaling pathway (Ramos and Fernandez-Sesma, 2015), JAK-STAT signaling pathway (Schneider et al., 2014), and cytokine-cytokine receptor interaction (Farahani et al., 2022). Meanwhile, the downregulated genes were associated with cellular energy metabolism and and cell cycle, such as Foxo signaling pathway (Zhang et al., 2005) and Apelin signaling pathway (Mlyczyńska et al., 2020) (**Figure 5B**). Analysis of the expression changes of genes in key signaling pathway indicated that it could upregulate the expression of certain genes related to immunity, such as IRF3 (Al and Brady, 2022), SOCS3 (Carow and Rottenberg, 2014), and IL32 (Zhu et al., 2022) (**Figure 5C**). Furthermore, two additional compounds have demonstrated comparable bioactivity, each exerting regulatory control over the expression profiles of numerous genes in A549 cells (Curcumin upregulated 3297 genes, downregulated 3541 genes, and Cardamonin upregulated 4374 genes, downregulated 4371 genes) (**Figures 5D, G**). Meanwhile, these genes upregulated by Curcumin and Cardamonin also included molecules related to intracellular immunity signaling pathway (**Figures 5E, F, H, I**). The above results indicated that these three compounds could exert certain antiviral effects by modulating the signaling pathways related to cellular immune responses.

## 4 Discussion

In this study, we have employed a GFP-based reporter influenza A virus (GFP-IAV) as a strategic tool to screen for potential anti-influenza compounds, leveraging the system’s capacity for high-throughput screening and instantaneous monitoring. The incorporation of GFP into the viral genome allows for the direct observation of viral replication, providing a rapid and quantifiable means to assess the antiviral potency of test compounds. This technique presents a significant improvement over conventional plaque assays or terminal assay methodologies, which are not only time-consuming but also necessitate post-infective processing. Utilizing the GFP-IAV, we have successfully identified three antiviral compounds from traditional anti-influenza Mongolian formulations.

Green Fluorescent Protein (GFP) offers the distinct advantage of enabling researchers to observe and track proteins in living cells in real time without disrupting cellular function, thanks to its inherent fluorescence (Tsien, 1998). GFP’s remarkable stability and non-toxic nature allow for its integration into a wide range of biological systems, facilitating diverse applications in scientific studies. Moreover, its capacity to form a chromophore autonomously, without the need for additional substrates or enzymes, makes GFP an exceptionally versatile and user-friendly tool in molecular and cellular biology (van Thor and Champion, 2023; Wang. et al., 2023). Fluorescent reporter viruses are currently widely used in the field of virology. To illustrate, a stable ZIKV GFP-reporter virus system with improved GFP visibility and stability was used for screening Zika virus inhibitors (Zhang. et al., 2021). There have also been reports of the application of GFP-reporter viruses for Dengue (Leardkamolkarn et al., 2012), West Nile viruses (Pierson et al., 2005) and some other viruses. The GFP reporting system is not the first time being used for the influenza A virus. Previous research has involved linking the GFP reporter gene to IAV NS segments (Perez et al., 2013) or NP segments (Antony and Schaeffer, 2013). The current antiviral drugs for IAV primarily targeted the neuraminidase (NA) of the virus, such as zanamivir (Hayden. et al., 2000) and oseltamivir (M, 2004). Therefore, attaching GFP to the NA segment could facilitate a more direct screening for compounds effective against NA. This is the reason we have opted to incorporate GFP into the NA segment. So, in our research, we have integrated GFP into the influenza virus NA segment via a 2A peptide linkage, which does not interfere with the normal infection and replication processes of the influenza virus. This modification allows the virus to express GFP during infection, and in the early stages of infection, the brightness of GFP fluorescence is directly proportional to the virus’s replication capacity. Using the reported virus, we have successfully identified three compounds with effective antiviral activity from a selection of more than twenty compounds.

The research status of antiviral properties in traditional Mongolian medicine is an area of growing interest. Studies have focused on classic Mongolian prescriptions known for antipyretic and antiviral effects, building a database of candidate compounds for further investigation (Yu et al., 2020). Bibliometric analyses have been employed to understand research trends and the current state of traditional Mongolian medicine, highlighting its potential in contemporary medicine (Kim and Kang, 2021). Pharmacological studies aim to provide modern interpretations of the traditional efficacy of Mongolian materia medica, contributing to the body of knowledge on its antiviral applications (Qu et al., 2022). Screening for effective antiviral compounds from traditional Mongolian medicine will also contribute to understanding the mechanisms of Mongolian medicine from a modern scientific perspective. Kaempferide is a naturally occurring flavonoid compound that is found in abundance in plants such as Scutellaria baicalensis, Andrographis paniculata, and Schizonepeta tenuifolia (Ren et al., 2019). Curcumin is abundantly found in the ginger family plant turmeric, which is also its main active ingredient (Weng and Goel, 2022). Turmeric is widely used to treat various inflammatory conditions and digestive system issues (Unlu et al., 2016). Cardamonin is also a naturally occurring flavonoid compound primarily found in plants of the ginger family, including white cardamom and grains of paradise. Its pharmacological activities typically include anti-inflammatory, antioxidant, and anti-tumor properties (Kim et al., 2015). Our research confirms that these three compounds effectively inhibit the proliferation of the influenza virus, enriching the study of antiviral mechanisms in traditional medicine.

The primary mechanisms of traditional Mongolian medicine in combating the IAV involve multiple aspects, including direct antiviral activity and modulation of the host’s immune response (Ma et al., 2023). Antiviral activity primarily includes preventing the binding of influenza virus to receptors on the host cell surface and inhibiting key enzymes such as the virus’s RNA polymerase to block viral replication. Regulating the host immune response primarily involves activating immune cells or increasing the production of antiviral cytokines to enhance the body’s immune response to viruses. Transcriptome sequencing results suggest that the inhibitory effects of these three compounds on the influenza virus may primarily be achieved by regulating the immune response signaling pathways within host cells, enhancing the cell’s natural antiviral immunity, and attenuating cell cycle activities. Past findings have suggested that the division of host cells promotes the accumulation of influenza A virus (IAV) proteins and the production of the virus (He et al., 2010; Ma et al., 2023). This suggests that the antiviral effects of these three compounds could be broad-spectrum, which is worth further verification.

However, modified viruses do not alter the fundamental processes of viral entry and infection in host cells, the introduction of fluorescent genes can sometimes compromise their replication efficiency. This reduced replication capacity, when compared to wild-type viruses, may skew the results obtained from high-throughput screening of antiviral compounds. The discrepancy arises because compounds might appear to inhibit the replication of the reporter virus more effectively than they would inhibit a wild-type virus, potentially resulting in the identification of false positives compounds that seem effective in the assay but may not be as effective in a clinical setting against the actual virus. Moreover, the fluorescence signal itself, which is used as an indicator of viral replication, can be influenced by certain drugs or experimental conditions. For example, some compounds might quench or enhance the fluorescence independent of their antiviral activity, or certain experimental conditions might affect the stability of the fluorescent protein (Ke and Sung, 2023), leading to fluctuations in signal intensity that do not correlate with actual changes in viral replication levels. Therefore, it is crucial to conduct follow-up experiments to validate the initial screening results.

In summary, by incorporating the GFP gene into the influenza virus NA segment, we successfully rescued GFP-tagged influenza A virus (GFP-IAV) using reverse genetics. Utilizing the characteristic that the intensity of GFP fluorescence is directly proportional to viral proliferation during GFP-IAV infection, we identified three natural compounds with anti-influenza virus activity from traditional Mongolian herbal formulas. Preliminary investigations into the antiviral mechanisms of these compounds were conducted using transcriptome sequencing, suggesting that they may exert their antiviral effects by modulating the host cell’s innate immune response signaling pathways. Although the use of fluorescent reporter viruses has certain limitations, their high throughput and efficiency offer significant potential in the research of antiviral drug screening.

## Data availability statement

The transcriptomic sequencing data presented in the study are deposited in the China National Microbiology Data Center (NMDC; https://nmdc.cn/en) repository, BioProject accession number NMDC10018695. The original contributions presented in the study are included in the article/**Supplementary Material**, further inquiries can be directed to the corresponding authors.

## Author contributions

MN: Data curation, Formal analysis, Writing – original draft. XL: Methodology, Validation, Writing – review & editing. SZ: Formal analysis, Resources, Writing – original draft. DZ: Formal analysis, Writing – original draft, Data curation. YC: Writing – original draft, Methodology. DP: Project administration, Writing – review & editing. TJ: Methodology, Supervision, Writing – review & editing. JS: Methodology, Writing – review & editing. JL: Conceptualization, Supervision, Writing – review & editing.
