# Supplementary material for: Screening for anti-influenza virus compounds from traditional Mongolian medicine by GFP-based reporter virus
Source: Front Cell Infect Microbiol. 2024 Jul 12;14:1431979. doi: 10.3389/fcimb.2024.1431979 (PMC11272615; doi:10.3389/fcimb.2024.1431979)
Supplement: Supplementary file 1 [file DataSheet_1.docx]

Supplementary Material

# Supplementary Figures and Tables

## Supplementary Figure

**Supplementary Figure 1.**

**(A)** Proliferative abilities of GFP-IAV and wild-type IAV in MDCK cells. **(B)** RNA copy numbers were measured by qRT-PCR at different time points (12, 18, 24 and 48 h post-infection) in supernatant of MDCK cells infected with GFP-IAV or wild-type IAV. **(C)** NP protein expression was detected by western blot at different time points (6, 12, 18, 24 and 48 h post-infection) in MDCK cells infected with GFP-IAV or wild-type IAV.


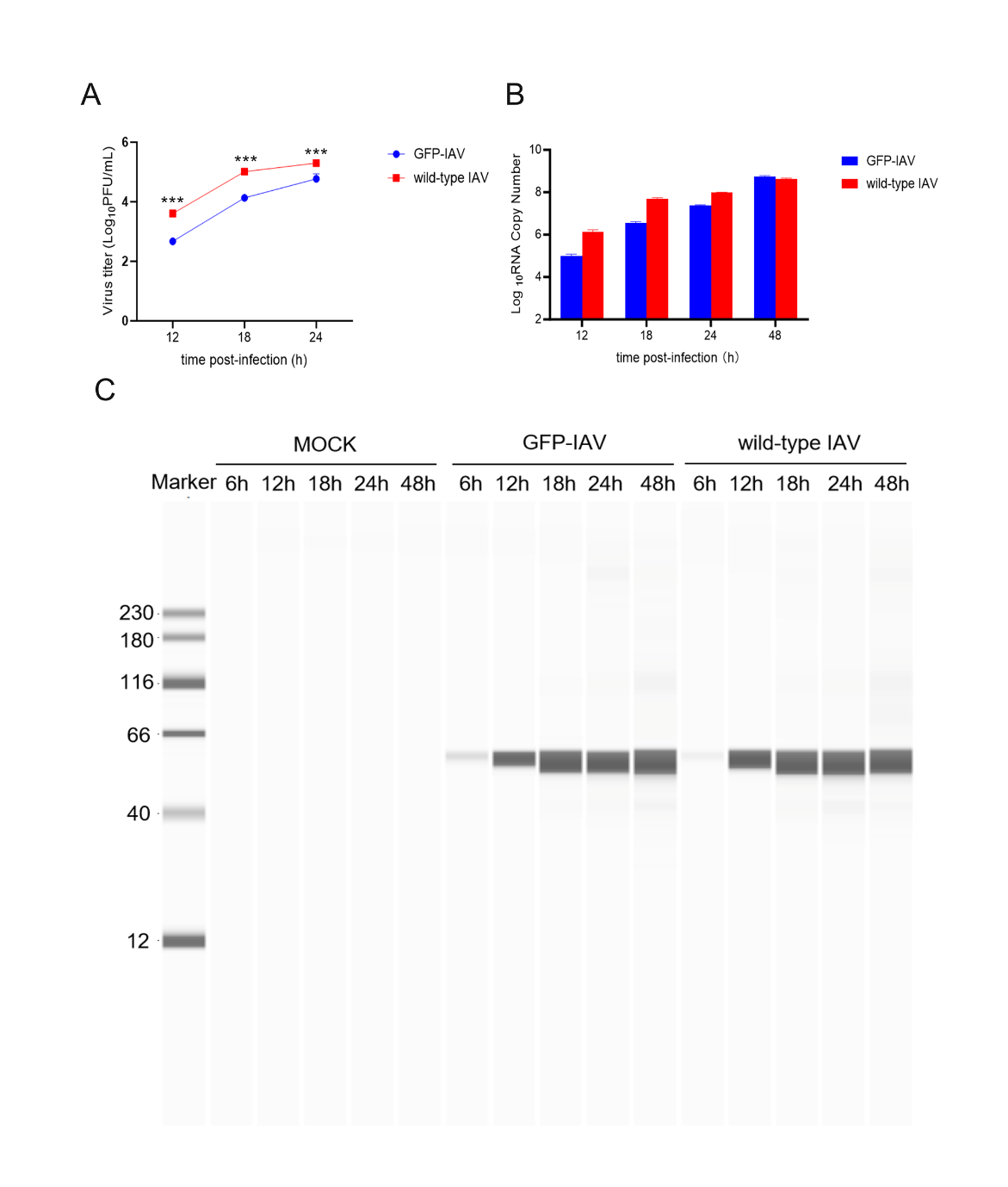


**Supplementary Figure 2.**

**(A)** CC_50_ values of three compounds. **(B)** IC_50_ values of three compounds.


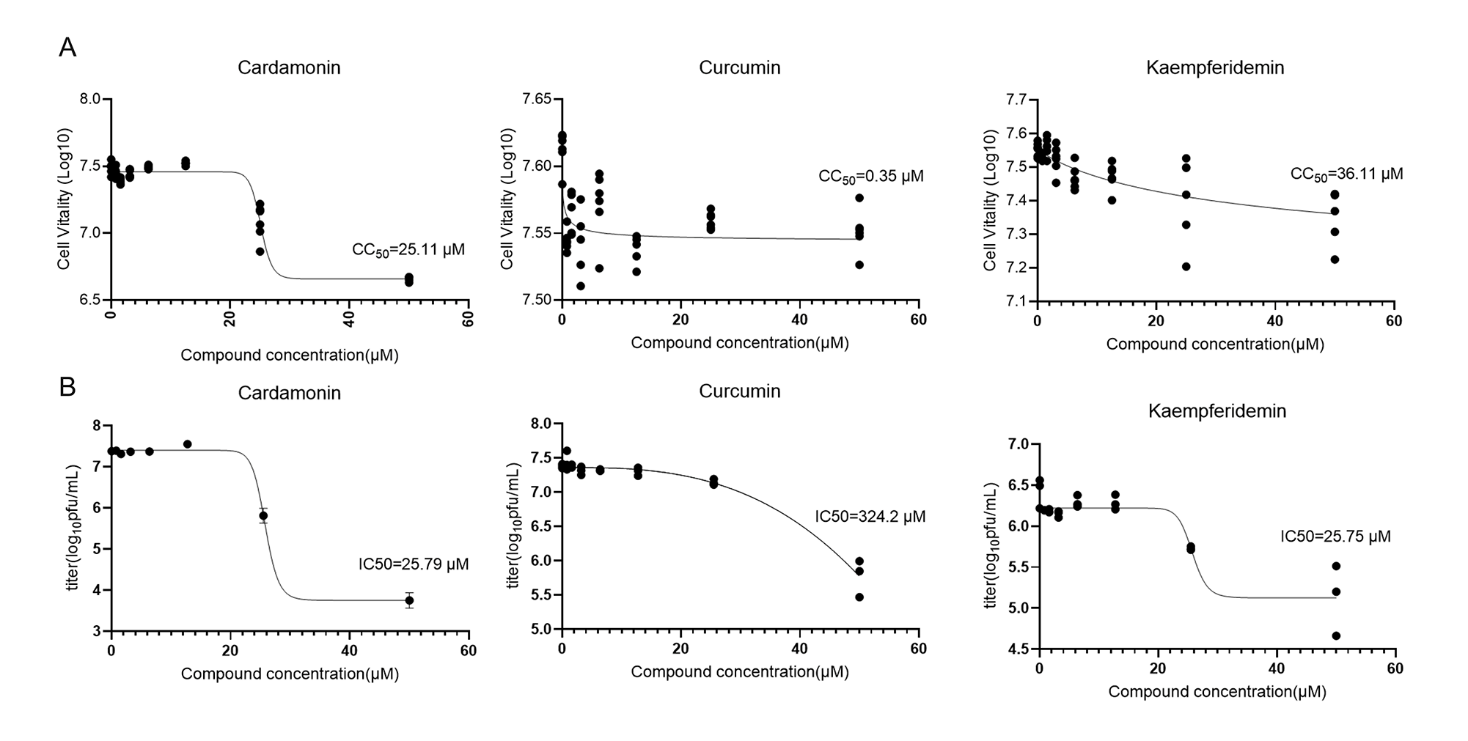


## Supplementary Table

**Supplementary Table 1.** **20 candidate compounds.**

| **Compounds** | **traditional Mongolian medicine** |
| --- | --- |
| Epigoitrin | Isatis indigotica |
| Coumarin | Radix Glehniae |
| Curcumol | Curcuma zedoaria |
| Limonin | Citrus aurantium |
| Cynarin | Cynara scolymus |
| Sophocarpine | Sophora flavescens |
| Sophoranol | Sophora flavescens |
| Hyperoside | Hypericum perforatum |
| Kaempferide | Scutellaria baicalensis |
| Artemitin | Artemisia annua |
| Isomangiferin | Pyrrosia Leaf |
| Picroside II | Picrorhiza kurrooa |
| Carvacrol | Origanum vulgare |
| Cardamonin | white cardamom |
| Curcumin | turmeric |
| Cynaroside | Lonicera japonica Thunb. |
| Isoliquiritigenin | Glycyrrhiza glabra |
| 3-Methoxyflavone | Artemisia incanescens |

**Supplementary Table 2.** CC_50_, IC50 and SI values of three compounds.

| Compounds | Index | A/California/California/07/2009 |
| --- | --- | --- |
| Cardamonin | CC50 (μM)  IC50 (μM)  SI (μM) | 25.11  25.79  0.97 |
| Curcumin | CC50 (μM)  IC50 (μM)  SI (μM) | 0.35  324.2  0.0011 |
| Kaempferide | CC50 (μM)  IC50 (μM)  SI (μM) | 36.11  25.75  1.40 |
